# Molecular Epidemiology of Multi-Drug Resistant *Pseudomonas aeruginosa* Isolates from Hospitalized Patients in Greece

**DOI:** 10.3390/microorganisms8111652

**Published:** 2020-10-24

**Authors:** Olga Pappa, Anastasia Maria Kefala, Kyriaki Tryfinopoulou, Marios Dimitriou, Kostas Kostoulas, Chrysa Dioli, Eleni Moraitou, Maria Panopoulou, Evaggelos Vogiatzakis, Athena Mavridou, Alex Galanis, Apostolos Beloukas

**Affiliations:** 1 Department of Biomedical Sciences, University of West Attica, 12243 Egaleo, Greece; akefala@uniwa.gr (A.M.K.); mardimitriou7@gmail.com (M.D.); cdioli@uniwa.gr (C.D.); amavridou@uniwa.gr (A.M.); 2 Central Public Health Laboratory, National Public Health Organization, 16672 Athens, Greece; k.tryfinopoulou@eody.gov.gr; 3 Laboratory of Microbiology, ‘Sotiria’ General Hospital, 152 Mesogeion Avenue, 11527 Athens, Greece; kostas.kostoulas@hotmail.com (K.K.); elenliamor@gmail.com (E.M.); vogia2@gmail.com (E.V.); 4 Laboratory of Microbiology, School of Medicine, Democritus University of Thrace, Dragana, 68100 Alexandroupolis, Greece; mpanopou@med.duth.gr; 5 Department of Molecular Biology and Genetics, Health Science School, Democritus University of Thrace, 68100 Alexandroupolis, Greece; agalanis@mbg.duth.gr; 6 Institute of Infection & Global Health, University of Liverpool, Liverpool L69 7BE, UK

**Keywords:** *P. aeruginosa*, multi drug resistance, oprD, DLST, HAIs, FEPR-CAZ_S_

## Abstract

Resistant *Pseudomonas aeruginosa* isolates are one of the major causes of both hospital-acquired infections (HAIs) and community-acquired infections (CAIs). However, management of *P. aeruginosa* infections is difficult as the bacterium is inherently resistant to many antibiotics. In this study, a collection of 75 *P. aeruginosa* clinical isolates from two tertiary hospitals from Athens and Alexnadroupolis in Greece was studied to assess antimicrobial sensitivity and molecular epidemiology. All *P. aeruginosa* isolates were tested for susceptibility to 11 commonly used antibiotics, and the newly introduced Double Locus Sequence Typing (DLST) scheme was implemented to elucidate the predominant clones. The tested *P. aeruginosa* isolates presented various resistant phenotypes, with Verona Integron-Mediated Metallo-β-lactamase (VIM-2) mechanisms being the majority, and a new phenotype, FEP_R_-CAZ_S,_ being reported for the first time in Greek isolates. DLST revealed two predominant types, 32-39 and 8-37, and provided evidence for intra-hospital transmission of the 32-39 clone in one of the hospitals. The results indicate that DLST can be a valuable tool when local outbreaks demand immediate tracking investigation with limited time and financial resources.

## 1. Introduction

Resistant *Pseudomonas aeruginosa* isolates are one of the major causes of hospital-acquired infections (HAIs) and community-acquired infections (CAIs). In 2018, the European Antimicrobial Resistance Surveillance Network (EARS-Net) reported that the percentage of carbapenem-resistant *P. aeruginosa* strains reached 38% in Europe [1]. Morbidity and mortality attributable to *P. aeruginosa* resistant strains are high and are considered poor prognosis markers [2]. Hospitalized patients are prone to bacterial infections upon admission or during hospitalization [2]. On the other hand, CAIs are transmitted and develop outside the hospital, but demand hospitalization (e.g., pneumococcal pneumonia), or are clinically present within 48 h from hospital admission, regardless of the initial cause of hospitalization (e.g. chickenpox) [3].

Management of *P. aeruginosa* infections still remains a clinical challenge as the bacterium is inherently resistant to many antibiotics [4]. *P. aeruginosa* is characterized as antibiotic resistant, since it demonstrates all known enzymatic (low outer membrane permeability/oprD loss, chromosomally encoded AmpC, as well as an extensive efflux pump system) and genetic mechanisms of resistance [5,6]. Due to the wide dispersion of the bacterium in the environment [4,7], as well as in the endogenous flora of hospitalized patients, it is important to implement powerful molecular typing tools to elucidate its molecular epidemiology and assess dispersal patterns of resistant strains [4].

Several typing methods have been used to study the evolution and genetic heterogeneity of *P. aeruginosa* because it is characterized by high genetic diversity [8,9,10,11,12]. In recent years, the development of Whole Genome Sequencing has given rise to the study of Multi-Drug Resistant (MDR) *P. aeruginosa*’s molecular epidemiology [13,14], but this is a high cost method demanding specialized staff making it difficult for many laboratories to implement.

Double Locus Sequencing Typing (DLST) is a newly developed typing scheme, first reported in 2014, that uses partial sequencing of three highly variable loci: *ms172*, *ms217*, and oprD [15]. As the combination of two loci gave resolution results only slightly lower than the combination of the three loci, the final scheme proposed by the authors includes the use of only the two loci (*ms172* and *ms217)* [15]. However, a number of published articles have shown that approaching the population analysis using numerous genetic markers results in more reliable data [15,16]. Specifically, oprD has been successfully used as a genetic marker for the analysis of the *P. aeruginosa* population [17,18,19,20]. As Pirnay et al. state, the oprD gene has been proved extremely important as (a) it is implicated in carbapenem resistance, (b) its structure reveals evidence of recombination events between *P. aeruginosa* isolates, and (c) it provides variability (high number of alleles) and stability (narrow clonal complexes show identical oprD sequences) at the same time [17].

The new sequence-based scheme was compared to Multi Locus Sequencing Typing (MLST) in a number of clinical and environmental *P. aeruginosa* isolates, proving that when epidemiological and phylogenetic analyses are conducted at a local level, MLST can be replaced by DLST [21]. The online publicly available DLST database (http://www.dlst.org/Paeruginosa/) uses nucleotide sequences of the two loci (*ms172* and *ms217*) to define the DLST type [15]. The method is new, and thus, there are scarce published data regarding its implementation for both clinical and environmental isolates [11,12,15,21].

The aims of the present study were (a) to elucidate the predominant *P. aeruginosa* clones in Greek hospitalized patients using the newly introduced DLST scheme, (b) to study the resistant phenotypes of the clinical *P. aeruginosa* isolates recovered from two tertiary-care hospitals, and (c) to reveal the distribution of the obtained resistant phenotypes among the various DLST types and the local epidemiology in terms of resistant mechanisms. We aim to propose the implementation of the DLST scheme as an epidemiological tool when local outbreaks demand immediate investigation with limited financial resources.

## 2. Materials and Methods

### 2.1. Settings and Bacterial Isolates

This study was conducted in the Molecular Microbiology and Immunology Laboratory (Micro.Mol lab) at the Department of Biomedical Sciences, University of West Attica, in collaboration with the Microbiology Laboratoriess of (a) the General University Hospital of Alexandroupolis in northern Greece (H1), a 670-bed tertiary-care hospital and (b) Thoracic Diseases General Hospital Sotiria of Athens in Central Greece (H2), an 800-bed tertiary-care hospital, during a two-year period (01/2016–12/2017). The study included (a) 25 isolates from H1, all recovered from blood cultures of patients hospitalized in various wards and (b) 50 isolates from H2, recovered from various samples from patients dispersed in different wards (Table S1). The isolates were initially collected and identified as *P. aeruginosa* using standard biochemical tests from the microbiological laboratories of the two hospitals, and pure cultures were sent to the Micro.Mol Lab for further phenotypical and molecular testing. All isolates were molecularly screened for the *P. aeruginosa* 16s rRNA gene [22] and four of them were negative; thus, the final collection include 71 isolates. Two reference strains were used as controls: (a) a clinical control provided by HPA/NEQAS (the HPA External Quality Control Scheme, Sheffield, UK) and (b) the *P. aeruginosa* PAO1 (Collection of Institute Pasteur CIP104116, www.crbip.pasteur.fr, Paris, France).

### 2.2. Collection and Analysis of Clinical and Epidemiological Data

A Case Report Form (CRF) was used to retrospectively collect epidemiological, clinical, and microbiological data. These included demographics (gender and age), microbiological and clinical data (*P. aeruginosa* isolation date and site, clinical importance, underlying diseases), and epidemiological data (date of admission, duration and department of hospitalization, movements between departments, any invasive medical intervention, and history regarding previous contact with the health care system or use of antibiotics in the last six months). Τo describe each sample by person, time and place, and to interpret the molecular typing data in the specific epidemiologic content, we combined temporal and spatial hospitalization data of each patient with the DLST typing data, separately for the two hospitals, using Microsoft Excel software. For each patient, in a separate line, we marked his/her entire hospitalization period with different color for different hospitalization units and the day of *P. aeruginosa* isolation of a specific DLST type facilitating both the recognition of the possible origin of the infection, as well as the simultaneous or subsequent hospitalization in the same unit of patients with the same DLST type *P. aeruginosa* strain.

### 2.3. Ethical Considerations/Approval

This study was approved (42981—23/06/2020) by the Institution Review Board of the University of West Attica (Athens, Greece).

### 2.4. Isolation of Genomic DNA

*P. aeruginosa* genomic DNA was extracted using the Purelink Genomic DNA mini kit (ThermoFisher, Antisel, Thessaloniki) following the manufacturer’s instructions after 48-h growth in nutrient broth and nutrient agar.

### 2.5. Antibiotic Susceptibility Testing (AST)

All isolates were tested for susceptibility implementing the standard Disk Diffusion method [21] and applying 11 commonly used antibiotics belonging to four different classes: non-carbapenem beta-lactams: ceftazidime (CAZ; 30 μg), cefepime (FEP; 30 μg), piperacillin (PIP; 75 μg), ticarcillin (TIC; 75 μg), ticarcllin/clavulanate (TCC; 75 μg/10 μg), aztreonam (ATM; 30 μg); carbapenems: imipenem (IPM; 10 μg) and meropenem (MEM; 10 μg); aminoglycosides: amikacin (AN; 30 μg), tobramycin (TOB; 30 μg), gentamicin (GM; 30 μg); fluoroquinolones: ciprofloxacin (CIP; 5 μg) according to ‘The European Committee on Antimicrobial Susceptibility Testing’ (EUCAST) guidelines [23]. The interpretation of the antibiotic-resistant phenotypes and the classification of *P. aeruginosa* isolates as MultiDrug Resistant (MDR), Resistant (R) and Susceptible (S) were performed according to the interim standard definitions for acquired resistance [24]. A phenotypic test with EDTA for metallo beta-lactamase (MBL) production was performed [25], and a new resistant phenotype (FEP_R_- CAZ_S_) was observed and evaluated [26,27,28].

### 2.6. Screening for Acquired Resistant Genes

All CARB_R_/EDTA (+) isolates were screened for the presence of three MBL genes, bla_VIM-2_, bla_IMP_, and bla_NDM_, as well as for bla_OXA-48_ carbapenemase [29]. The FEP_R_- CAZ_S_ isolates were subjected to PCR for the presence of bla_OXA group I_, bla_PSE-1_, and bla_OXA group III_ beta lactamases based on previous studies [26,27,28]. All 71 isolates were screened for the presence of mcr genes (1–5) following the published protocol of Rebelo A.R. et al. [30]. Control isolates for the five positive *mcr*-genes were kindly provided by the Technical University of Denmark [30].

### 2.7. Molecular Typing

#### 2.7.1. Double-Locus Sequence Typing and oprD-Typing

DLST was implemented across 73 isolates of *P. aeruginosa* (71 isolates plus the two reference strains, PAO1 and NEQAS), with oprD-typing in 63 isolates (62 isolates plus the reference strain PAO1), as nine isolates did not express the oprD gene [12,15]. If no sequence of good quality was obtained after the second step, the result for the isolate was considered a null allele [15]. DLST sequences were subjected to the DLST database (http://www.dlst.org/Paeruginosa/) for allele assignment of the genetic markers *ms172* and *ms217*; if there was no identification for the submitted locus, the procedure for the submission of new alleles in the DLST database was followed and a new locus number was assigned [12,15]. To assess the variations in the oprD gene, the oprD sequences of the IPM_R_-MEM_R,_ IPM_S_-MEM_S,_ IPM_R_-MEM_S,_ and IPM_S_-MEM_R_ isolates were compared to the oprD sequence of the reference strain PAO1 (Ref. seq.: NC_002516.2_P.aer_PAO) using ClustalX2 multiple alignment (http://www.clustal.org/) and MEGA v.7.

#### 2.7.2. DLST and oprD Data Analysis

DLST data were analyzed using Global optimal eBURST analysis ([31] http://www.phyloviz.net/goeburst/accessed on 01/08/2019) as previously described using a similar approach [12]. Maximum likelihood (ML) phylogeny of the oprD-gene was assessed with RaxML-HCP2 v8 [30] using GTR + I + G that was identified as the best-fitted model using jModelTest2 [32,33]. During the analysis, the Ref. seq.: NC_002516.2_P.aer_PAO was used to align the sequences. The discriminatory power (D) of the methods was calculated using the online tool ‘Discriminatory Power calculator’ available at http://insilico.ehu.es/mini_tools/discriminatory_power/index.php.

## 3. Results

### 3.1. Study Population and Characteristics

Overall, the studied *P. aeruginosa* isolates collected from 71 hospitalized patients (24 in H1 and 47 in H2) with a mean age of 69.54 ± 16.88 years and 40 (56.3%) were male. In particular, in H1, all 24 *P. aeruginosa* isolates were recovered from blood cultures of patients with a mean age of 65.41 ± 19.18 years and 12 (50%) males. The patients stayed in an Intensive Care Unit (ICU) and/or medical and surgical departments with recorded movements for nine patients. The mean length of hospitalization until the *P. aeruginosa* isolation was 19.40 ± 21.53 days, and the total length of stay was 35.92 ± 32.20 days. In H2, the 47 *P. aeruginosa* isolates were recovered from various clinical specimens of equal numbers of patients with a mean age of 71.56 ± 15.47 years and 28 (59.6%) of male sex. The patients were hospitalized in ICU and/or medical departments with recorded internal movements for 33 of them. The mean length of hospitalization until the *P. aeruginosa* isolation was 18.72 ± 27.98 days, and the total length of stay was 62.43 ± 37.11 days.

### 3.2. Antimicrobial Susceptibility Profiles and Detection of Resistant Genes

**Figure 1:**
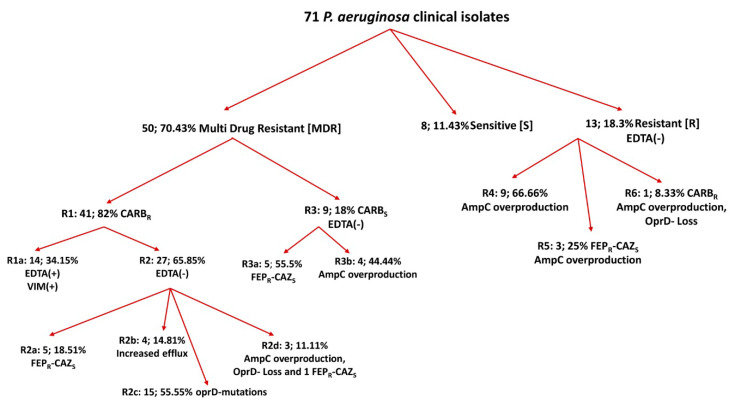
Resistant profiles of the 71 clinical *P. aeruginosa* isolates.

The 71 *P. aeruginosa* isolates presented various resistant phenotypes (presented in Figure 1, as well as in the heatmap in Table S4). Thirty-three CARB_R_ (Carbapenem Resistant) isolates from H2 were isolated from various clinical samples, while the eight CARB_R_ isolates from H1 were from blood cultures (Table S1). The EDTA double synergy test was positive in 14 CARB_R_ isolates from both hospitals (CARB_R_ –EDTA (+) isolates). All 14 PCR amplicons were sequenced for VIM-2 Metallo Beta Lactamase and were negative for other MBL genes tested, bla_IMP_ and bla_NDM_, and for the presence of bla_OXA-48_ carbapenemase (Table S1). In total, 6/14 CARB_R_–FEP_R_ –CAZ_S_ isolates presented a resistant profile [CAZ]_S_-[ATM-FEP]_R_-[IPM]_R_ with possible oprD loss (Figure 1, Table S4, R2a, R2d), and 5/14 CARB_S_ –FEP_R_ –CAZ_S_ presented a resistant profile [CAZ]_S_-[ATM-FEP]_R_-[IPM]_S_ with induction of AmpC beta-lactamase and resistance to quinolones (Figure 1, Table S4, R3a), which seem to be good substrates for different efflux pumps [28]. The remaining 3/14 FEP_R_ –CAZ_S_ were assigned to the R5 resistant profile (Figure 1, Table S4) All were negative for the presence of bla_OXA group I_, bla_PSE-1_, and bla_OXA group III_ beta lactamases (Table S1). Finally, all 71 tested isolates were negative for the five mcr-genes tested.

### 3.3. Molecular Typing

#### 3.3.1. Double-Locus Sequence Typing

**Figure 2:**
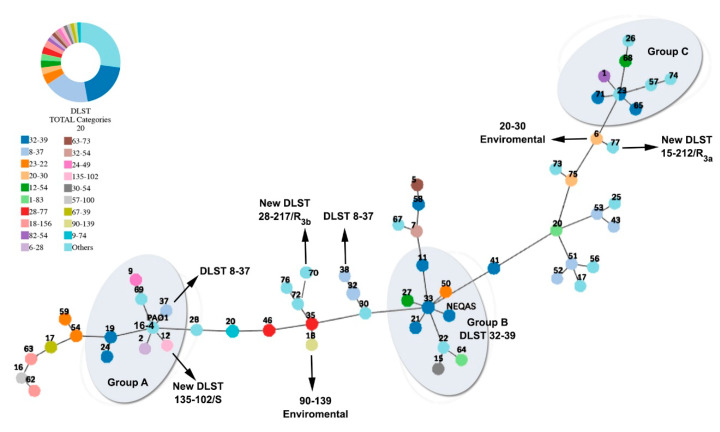
Minimum spanning tree resulting from e-burst analysis of *P. aeruginosa* isolates based on the DLST data. The 35 DLST-types are presented here, with the predominant and the new ones in bold.

Seventy-three isolates (including the two reference strains) were successfully typed with the DLST scheme (typeability = 100%). DLST was able to assign an already known allele number to the 68 isolates, while for the isolates (ID 12, 70, 77 in Table S1), three new loci were recognized (ID: 12 *ms172* allele 135, ID 70 *ms217* allele 217, ID 77 *ms217* allele 212 http://www.dlst.org/Paeruginosa/ms172.txt). eBURST analysis (implementing a 90% cut-off) revealed 35 types in total, with DLST types 8–37 (13/71;18.3%) and 32–39 (13/71; 18.3%) being the predominant ones. The reference strains NEQAS and PAO1 belonged to the DLST types 32–39 and 16–4, respectively (Figure 2). There were six DLST types (1–83, 12–54, 18–156, 20–30, 23–22, and 28–77) with two or three isolates each; the remaining 27 isolates presented as singletons [i.e., the new DLST type 135–102 (the new *ms172* allele was combined with only one *ms217* loci) and the two newly found DLST types 28–217 and 15–212 (the two new *ms217* loci were combined with two already assigned *ms172* alleles] (Table S1; Figure 2). The discriminatory power of the method is considered high (D = 0.93), as it was able to distinguish genetically close isolates among different DLST types (Figure 2, GROUP-A). The predominant DLST type 8–37 was highly dispersed in the eburst tree (Figure 2) and it was associated exclusively with patients from H2 who deal with severe respiratory problems and they had received antibiotics in the previous six months (Table S1). The 8–37 isolates were collected from various specimens and characterized as MDR distributed among three resistant phenotypes R2a, R2c, and R1a (Table S1; Figure 1). The DLST type 32–39 was characterized by lower diversity (Figure 2, GROUP-B and -C) and it was associated almost equally with patients from both hospitals. The VIM-2 phenotype was associated mainly with the predominant DLSTs (8–37: 7/14; 32–39: 4/14) and with three singletons; the new FEP_R_–CAZ_S_ phenotype was scattered throughout the phylogenetic tree (Table S1; Figure 2). The new DLST types appeared as one sensitive and two CARB_S_ isolates. For the six DLST types which included two or three isolates and the 27 singletons there was no significant correlation with the obtained resistant phenotypes.

#### 3.3.2. oprD Typing

**Figure 3:**
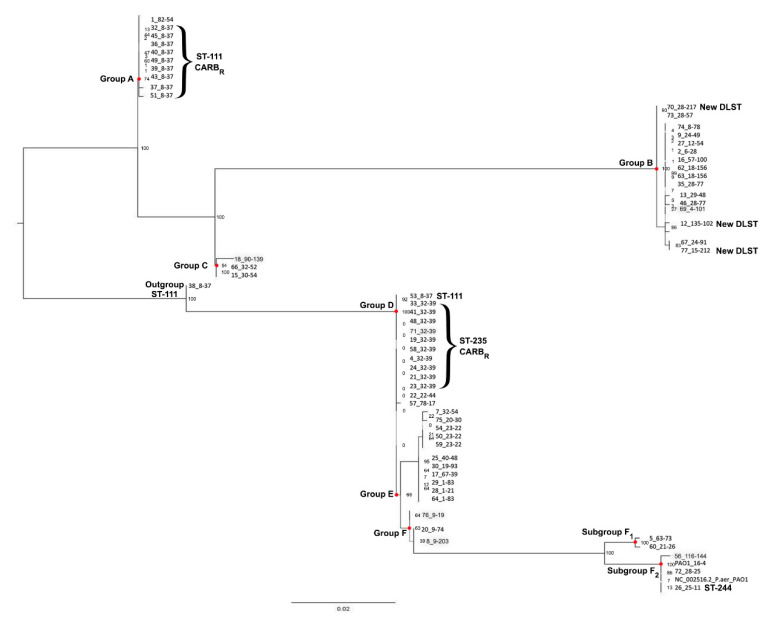
ML phylogeny of the *P. aeruginosa* isolates based on the 62 oprD sequences of the tested isolates; the Ref. seq.: NC_002516.2_P.aer_PAO was used to align the sequences. Correlation of the oprD-groups with the DLST-types is also presented.

The ML analysis revealed six major clusters: A, B, C, D, E, F- two sub-groups (F1–-F2) and one out-group (isolate 38) (Table S1; Figure 3). The discriminatory power of the method is considered high (D = 0.84), as it was able to distinguish genetically close isolates among different oprD-groups (Figure 3).

The predominant DLST type 32–39 isolates, all CARB_R_, were grouped into oprD-group D and group A, presenting a low degree of divergence between sequences, (Table S1; Figure 3). The DLST type 8–37 isolates, all CARB_R_ as well, had more variable oprD sequences, as the majority was grouped in oprD–group A but with a higher degree of divergence between sequences. Οne isolate (38) was characterized as an out-group, and isolate 53 was placed in the oprD–group D (Table S1; Figure 3). The remaining DLST types correlated with many different oprD–groups and resistant phenotypes (Table S1; Figure 3).

The comparative analysis of the oprD sequences revealed a high diversity, especially in IPM_R_-MEM_R_ and IPM_S_-MEM_S_, whilst the IPM_R_-MEM_S_ and IPM_S_-MEM_R_ isolates had relatively fewer mutations (Table S2).

Among the 31 IPM_R_-MEM_R_ isolates, there was one isolate with an insertion of ten bases at site 124 (isolate 29; Table S2), two isolates with an insertion of 11 bases at site 483 (isolates 62 and 63; Table S2), and a one-base insertion in isolate 17 (Table S2), which was enough to separate it from the reference strain PAO1. Small deletions (1–-3 bp) at various sites were observed in the rest of the isolates (Table S2). In relation to the DLSTs, it seems that 5/10 IPM_R_-MEM_R_ 32–39 isolates shared exactly the same stop codons (all belonging to the oprD–group D; 4, 21, 23, 24, 71; Table S2), and 2/10 isolates (isolates 41 oprD–group A and isolate 33 oprD–group D; Table S2) presented a different pattern including small deletions. Eight of the 13 IPM_R_-MEM_R_ 8-37 isolates shared the same deletions and stop codons (all belonging to the oprD–group A; 39, 36, 40, 32, 51, 49, 45, 43; Table S2).

Among the 29 IPM_S_-MEM_S_ isolates, 15 isolates (Table S3) presented the same 2 bp deletion (st 1127, 1157; Table S3). Eleven of them (11/15;67, 69, 70, 74, 77, 2, 9, 12, 13, 16, 27), all belonging to the oprD–group B with various DLST types, encoded incomplete oprD proteins due to the presence of a premature stop codon at the same positions (SC 190, 364, 588, 1102; Table S3). In the remaining 14/29, there was variability in the mutation events, and different patterns were observed (Table S3).

The IPM_R_-MEM_S_ isolate (73 with DLST 28–57; Table S1) had a 3 bp deletion at two sites and encoded incomplete oprD proteins due to the presence of premature stop codons at six different positions (deletion on 1127, 1157, 169 and SC on 190, 364, 589, 1045, 1075; Table S1). Finally, 3/4 IPMs-MEM_R_ isolates, all DLST 32–39 and the oprD group D, presented the same SC profile (19, 48, 58; SC 361, 691; Table S1), while isolate 56, DLST 116–144 and oprD group F2 encoded an incomplete oprD protein due to the presence of premature stop codons in two different positions (SC 364, 1051; Table S1).

### 3.4. Spatial and Temporal Mapping of the Main DLST Types in the two Hospital Settings

In H1, DLST 32–39 was isolated during a 3-month period (June–August 2016) from patients hospitalized mainly in the ICU and the Orthopedics department. This DLST type was first isolated from a patient hospitalized in the ICU and one month later from another ICU patient who had been transferred from the Orthopedics department the day before. Finally, two months since its first isolation, DLST 32–39 was isolated in two consecutive days from three patients hospitalized in the same Orthopedics department. In H2, DLST type 32–39 was recovered from seven patients during an 11-month period, with a cluster of five patients having been hospitalized in MAF or having overlapping hospital stays during a shorter, 5–month period. In this hospital, the predominant DLST type 8–37 was isolated from 13 patients hospitalized in medical departments, with 11 of them having been hospitalized in ICU during their hospital stay in two separate periods; November 2016–January 2017 (n = 8 patients) and March–April 2017 (n = 3 patients).

## 4. Discussion

To the best of our knowledge, this is the first time that an attempt has been made to elucidate the predominant *P. aeruginosa* clones in Greek hospitals using the newly proposed DLST scheme. The resistant phenotypes of the clinical *P. aeruginosa* isolates recovered from the two specific hospitals in Greece have not been studied before so thoroughly. The study also sought to consider the distribution of the obtained resistant phenotypes among the various DLST types and the local epidemiology in terms of resistant mechanisms.

The emergence of MDR *P. aeruginosa* strains is a well-characterized issue, and the literature is rich in relevant information [6,14,34,35,36,37]. Carbapenems are the most widespread antibiotics used in clinical practice to treat bacterial infections [38,39,40], and the resistance of *P. aeruginosa* isolates to these antibiotics is always a serious problem for the clinician. In the tested population, 82% of the MDR isolates were characterized as CARB_R_, being resistant to one or both of the carbapenems tested (Figure 1, Table S4). The high percentage of CARB_R_ isolates in Greece is not surprising as, according to WHONET data (The Greek System for the Surveillance of Antimicrobial Resistance; www.mednet.gr/whonet/) for the tested years regarding *P. aeruginosa* strains isolated from various clinical samples of the medical and surgical wards and the ICUs of hospitals in Greece, resistance to imipenem occurs at a high percentage, with ICUs and blood cultures standing out (http://www.mednet.gr/whonet/); these findings are consistent with what has been previously reported [2,41,42].

One of the most common acquired resistance mechanisms present is the production of metallo-beta-lactamase VIM. In total, 34.15% of the CARB_R_ isolates were characterized as VIM-2 producers which were identified exclusively from patients from Pneumology and ICUs in H2 (Table S1). The bacterium is known to colonize the lungs of patients with Cystic Fibrosis with highly resistant VIM-2 isolates [43], but it is also widespread in patients with other respiratory problems [44,45,46]. The isolation of MDR/VIM-2 strains from sputum and bronchial secretions during the present study was not surprising, although it was the first time that the *P. aeruginosa* population of H2 was tested for MBL production.

Additional evaluation of the obtained resistant profile according to relevant articles in the literature outlined a new phenotype in 5/27 isolates, the FEP_R_–CAZ_S_ (Figure 1, Table S4, R2a, R2d, R3a, R5 phenotypes), which was first reported in a *P. aeruginosa* strain isolated from a rectal sample and associated with the production of an oxasilinase (class D carbapenemase), bla_OXA-31_ [26]. Since then, this phenotype has been scarcely reported in *P. aeruginosa* clinical strains isolated from respiratory tracts and wounds, and in both cases, it was associated with an extensive efflux pump system (MexCD–-OprJ, MexAB–- OprM) and an Extended-Spectrum β-lactamase (integron based PSE-1 β-lactamase) [27,28], but it has never been reported in *P. aeruginosa* isolates from Greek hospitals. According to WHONET data, *P. aeruginosa* CAZ_R_ isolates are the ones that persist. In the present study, the FEP_R_–CAZ_S_ phenotype appeared in isolates mainly from clinical samples of patients in Pneumology Units in H2 and in isolates from blood cultures of a patient in H1 presenting the resistant profile [CAZ]_S_-[ATM-FEP]_R_-[IPM]_R/S_ (Table S1). None of the isolates produced positive results for the tested resistant genes indicated by the relevant articles in the literature [26,27,28]. The results are not surprising as (a) there is a significant number of OXA genes that could be implicated in these phenotypes [46,47] and (b) it seems that overproduction of chromosomal AmpC β-lactamase and efflux pump systems are the most common mechanisms responsible for the FEP_R_–CAZ_S_ phenotype [27,28]; in both scenarios, additional experiments have to be applied to elucidate the molecular mechanism harboring this specific phenotype.

The DLST markers are considered highly stable in the case of local phylogenetic studies; however, during a long-term investigation, they probably undergo genetic changes [15]. eBURST analysis of DLST data identified eight DLST types and 27 singletons providing additional evidence that *P. aeruginosa* is a non-clonal population undergoing significant recombination events resulting in strains with unique genetic characteristics [12,16]. The high diversity of the loci *ms217* we found is consistent with the DLST data base (*ms172* = 142 alleles vs. *ms217* = 228; http://www.dlst.org/Paeruginosa/; accessed on 20 July 2020).

Four out of five patients in H1 and five out of seven patients in H2, all infected with *P. aeruginosa* DLST type 32–39, were considered as close epidemiologically linked, thus DLST provided evidences for putative intra-hospital transmission. Similarly, DLST 8–37 was isolated from 13 patients hospitalized in different medical departments in H2, with 11 of them having overlapping hospitalizations in the same ICU unit. These findings, even in small numbers of the tested isolates, highlight the emergence of ‘high-risk’ clones in the specific hospitals, as DLST 32–39 has been related to ST–235, which is the major ST–clone responsible for many outbreaks worldwide harboring a number of ESBL– and MBL–resistant genes, and 8–37 has been related to ST–111 which is characterized as an MBL–producing endemic lineage [48,49,50]. It has been recently stated that ST–235/DLST 8–37 possess a unique combination of resistant genes that may have contributed to the ability of the clone to acquire mobile resistant elements among local populations [51].

The two predominant DLST types consisted mainly of VIM-2 producers presenting resistance to IPM which was the only carbapenemase found in the tested collection. Among the 27 singletons, there was one DLST type, 25–11, which appeared in a wild isolate from blood culture. The DLST type 25–11 has been correlated with ST–244, which is a known intercontinental MBL-producing clone and it has appeared mainly in isolates with the wild-type susceptibility phenotype in clinical and environmental *P. aeruginosa* isolates [52,53]. However, it has been found in VIM-2 producers in Greece and in other countries [41,54] and recently it has been associated with a colistin-resistant *P. aeruginosa* isolate co-harboring the bla_NDM_ [55].

The method managed to reveal three new DLST types, 135–10, 28–217, and 15–212, in one wild isolate and in two CARBs presenting other enzymatic resistant mechanisms, such as the first-appearing FEP_R_–CAZ_S_ (Figure 2, Table S1). Usually the new genotypes appear in wild isolates; the fact that in this study the new DLST types appeared in resistant isolates could be an indication that the specific resistant patterns favor significant mutations when the antibiotic pressure is high, resulting in new *ms172–*-*ms217* combinations [21]. According to the current published information it seems that the DLST types 90–139 and 20–30 rarely occur and they have not been related to any of the known MLST-types. Specifically, the 90–139 and 20–30 types have appeared before in *P. aeruginosa* isolates from aquatic habitats in Greece [12]. In the present tested clinical isolates, both DLST types were associated with oprD-loss and chromosomally encoded AmpC. DLST 20–30 has been previously reported in clinical *P. aeruginosa* isolates [15], while 90–139, as far as we know, has not been mentioned before in clinical isolates.

In the studied population, the imipenem resistance was due both to the production of bla_VIM-2_ and oprD mutational events (Figure 1, Table S4, R1a and R2c phenotypes), in contrast to other published studies, which state that the oprD gene was a major determinant of resistance to imipenem [56,57,58]. As expected, the results showed a high diversity in the oprD sequences among the IPM_R_-MEM_R_ strains resulting in high polymorphism in their genetic analysis. The majority of the mutational events were due to small or large deletions and to premature stops codons; it has been stated that premature stop codons occur mainly in IPM_R_ isolates [10]. The IPM_S_-MEM_S_ strains had fewer mutations compare to CARB_R_ isolates, but a wide variety of amino acid changes in the oprD-gene were detected here too, indicating that the loss of oprD-porin is not restricted to carbapenem-resistant clinical isolates [10,58]. The presence of deficient oprD-proteins in susceptible *P. aeruginosa* isolates has not been yet fully explained, although some authors have tried to give some answers [59,60].

Finally, this study was focused on *P. aeruginosa* isolates recovered from patients from two tertiary referral hospitals. In the near future we aim to enroll additional Greek hospitals (resulting in more clinical isolates) in the project, aiming at both a complete evaluation of the DLST scheme and a more complete molecular and epidemiological characterization of the population of the clinical *P. aeruginosa* isolates in Greece.

## 5. Conclusions

The emergence of MDR *P. aeruginosa* isolates has been thoroughly studied over the last 20 years with CARB_R_ phenotypes standing out. Our results further confirm this, as in the studied population, 82% of the MDR isolates were CARB_R_. The imipenem resistance observed was due both to the production of blaVIM-2 and oprD mutational events. However, new resistant phenotypes are constantly revealed such as the new phenotype, here called FEP_R_–CAZ_S_, which has never been reported in *P. aeruginosa* isolates from Greek Hospitals, although according to WHONET data, *P. aeruginosa* CAZ_R_ isolates are the ones that persist. eBURST analysis of the DLST data identified eight DLST types (including the two predominant ones 8–37 and 32–39) and 27 singletons among all 71 isolates. The high non-clonality in the studied isolates was mainly due to the high diversity of the loci *ms217.* It seems that DLST gave evidence of putative intra-hospital spread of the two predominant clones, DLST 32–39 and DLST 8–37. This fact, even in a small number of the studied isolates, highlights the emergence of ‘high-risk’ clones in the specific hospitals, as the 32–39 DLST has been related to ST–235, and 8–37 to ST–111. Finally, the majority of the mutational events in the IPM_R_-MEM_R_ isolates were due to small or large deletions and to premature stops codons, while the results from the oprD analysis of the IPM_S_-MEM_S_ isolates indicate that deficiency of the oprD-porin is not restricted to CARB_R_ clinical isolates.

## Supplementary Materials

The following are available online at https://www.mdpi.com/2076-2607/8/11/1652/s1, Table S1_Raw Data, Table S2_ Resistance profile and DLST types vs oprD analysis and oprD grouping for the 31 IPM_R_-MEM_R_ isolates, Table S3_ DLST types vs oprD analysis and oprD grouping for the 29 IPMs-MEM_s_ isolates, and Table S4_heatmap.
