# Supplementary material for: Molecular Epidemiology of Multi-Drug Resistant Pseudomonas aeruginosa Isolates from Hospitalized Patients in Greece
Source: Microorganisms. 2020 Oct 24;8(11):1652. doi: 10.3390/microorganisms8111652 (PMC7693957; doi:10.3390/microorganisms8111652)
Supplement: Supplementary file 1 [file microorganisms-08-01652-s001.zip › Tables S2 and S3.pdf]

Table S2. Correlation of the Resistance profile and the DLST types to the oprD analysis and oprD grouping for the 31 IPM_R-MEM_R isolates.

a/a of isolate	Resistance Profile	oprD-group	oprD-analysis	DLST	
39	R2c	A	deletion 1127, 1157 st (3 bases in each site), SC 514	8	37
37	R1a/VIM-2	A	deletion 900 st (1 base) and 1127, 1157 st (3 bases in each site), SC 514	8	37
36	R2c	A	deletion 1127, 1157 st (3 bases in each site), SC 514	8	37
40	R1a/VIM-2	A	deletion 1127, 1157 st (3 bases in each site), SC 514	8	37
32	R2a	A	deletion 1127, 1157 st (3 bases in each site), SC 514	8	37
38	R1a/VIM-2	Out-group	deletion 899 st (2 bases), SC 514	8	37
53	R1a/VIM-2	D	deletion 899 st (2 bases), SC 361, 664, 691	8	37
51	R2a	A	deletion 1127, 1157 st (3 bases in each site), SC 514	8	37
49	R1a/VIM-2	A	deletion 1127, 1157 st (3 bases in each site), SC 514	8	37
45	R2c	A	deletion 1127, 1157 st (3 bases in each site), SC 514	8	37
44	R1a/VIM-2	Loss		8	37
43	R2c	A	deletion 1127, 1157 st (3 bases in each site), SC 514	8	37
52	R1a/VIM-2	Loss		8	37
41	R1a/VIM-2	A	deletion st 159 (1 base), SC 361, 664, 691	32	39
33	R2c	D	deletion 899 st (2 bases), SC 361, 664, 691	32	39
65	R1a/VIM-2	Loss		32	39
4	R2c	D	SC 361, 664, 691	32	39
11	R2c	Loss		32	39
21	R2c	D	SC 361, 664, 691	32	39
23	R2c	D	SC 361, 664, 691	32	39
24	R2c	D	SC 361, 664, 691	32	39
71	R1a/VIM-2	D	SC 361, 664, 691	32	39
31	R1a/VIM-2	Loss		32	39
29	R2c	E	insertion 124 st (10 bases), SC 361, 664, 691	1	83
17	R2a	E	insertion 798 st (1 base), SC 361, 664, 691	67	39
35	R1a/VIM-2	B	deletion 1127, 1157 st (3 bases in each site), SC 190, 229, 364, 589	28	77
62	R2c	B	deletion 483 st (11 bases) and 1127, 1157 st (3 bases in each site), SC 190, 229, 364, 589	18	156
63	R2c	B	deletion 483 st (11 bases) and 1127, 1157 st (3 bases in each site), SC 190, 229,	18	156

			364, 589		
59	R1a/VIM-2	E	SC 361, 664, 691	23	22
46	R2d	B	deletion 900 st (1 base) and 1127, 1157 st (3 bases in each site), SC 190, 229, 364, 589	28	77
50	R1a/VIM-2	E	SC 361, 664, 691	23	22

Table S3. Correlation of the DLST types to the oprD analysis and oprD grouping for the 29 IPMs-MEM$_s$ isolates (Resistance profile: all isolates were sensitive to the antibiotics tested).

a/a of isolate	oprD-group	oprD-analysis	DLST	
66	C	deletion 1126, 1157 st (2 bases), SC 514, 799, 961, 1102	32	52
18	C	deletion 1126, 1157 st (2 bases), SC 514, 799, 961, 1102	90	139
15	C	deletion 1126, 1157 st (2 bases), SC 514, 799, 961, 1102	30	54
67	B	deletion 1126, 1157 st (2 bases), SC 190, 364, 588, 1102	24	91
69	B	deletion 1126, 1157 st (2 bases), SC 190, 364, 588, 1102	4	101
70	B	deletion 1126, 1157 st (2 bases), SC 190, 364, 588, 1102	28	217
74	B	deletion 1126, 1157 st (2 bases), SC 190, 364, 588, 1102	8	78
77	B	deletion 1126, 1157 st (2 bases), SC 190, 364, 588, 1102	15	212
2	B	deletion 1126, 1157 st (2 bases), SC 190, 364,588, 1102	6	28
9	B	deletion 1126, 1157 st (2 bases), SC 190, 364, 588, 1102	24	49
12	B	deletion 1126, 1157 st (2 bases), SC 190, 364, 588, 1102	135	102
13	B	deletion 1126, 1157 st (2 bases), SC 190, 364, 588, 1102	29	48
16	B	deletion 1126, 1157 st (2 bases), SC 190, 364, 588, 1102	57	100
27	B	deletion 1126, 1157 st (2 bases), SC 190, 364, 588, 1102	12	54
28	E	SC 229, 361, 664, 691, 799, 919, 961, 1129	1	21
75	E	SC 229, 361, 664, 691, 799, 919, 961, 1129	20	30
30	E	insertion 131 st (10 bases), SC 229, 361, 664, 691, 799, 919, 961, 1129	19	93
54	E	SC 229, 361, 664, 691, 799, 919, 961, 1129	23	22
7	E	SC 229, 361, 664, 691, 799, 919, 961, 1129	32	54
25	E	SC 229, 361, 664, 691, 799, 919, 961, 1129	40	48
76	F	SC 229, 361, 664, 691, 799, 919, 961, 1129	9	19
8	F	SC 229, 361, 664, 691, 799, 919, 961, 1129	90	139
20	F	SC 229, 361, 664, 691, 799, 919, 961, 1129	9	74

72	F2	SC 229, 364, 664, 799, 919, 1129	28	25
26	F2	SC 229, 364, 664, 799, 919, 1129	25	11
57	D	SC 229, 361, 664, 691, 799, 919, 961, 1129	78	17
60	F1	SC 229, 310, 664, 799, 919, 961, 1129	21	26
5	F1	SC 229, 310, 361, 664, 799, 919, 1129	63	73
1	A	deletion 1126, 1157 st (2 bases), SC 229, 514, 799, 961	82	54
